# Sex Differences in the In Vivo Exposure Process of Multiple Components of *Gelsemium elegans* in Rats

**DOI:** 10.3390/metabo13010033

**Published:** 2022-12-24

**Authors:** Meng-Ting Zuo, Meng-Die Gong, Xiao Ma, Wen-Bo Xu, Zi-Yuan Wang, Mo-Huan Tang, Yong Wu, Zhao-Ying Liu

**Affiliations:** 1 College of Veterinary Medicine, Hunan Agricultural University, Changsha 410128, China; 2 Hunan Engineering Technology Research Center of Veterinary Drugs, Hunan Agricultural University, Changsha 410128, China

**Keywords:** *Gelsemium elegans*, toxicity, absorption, metabolism, distribution, gelsenicine

## Abstract

Asian *Gelsemium elegans* (*G. elegans*) has a wide range of pharmacological activities. However, its strong toxicity limits its potential development and application. Interestingly, there are significant gender differences in *G. elegans* toxicity in rats. This work aimed to elucidate the overall absorption, distribution, metabolism, and excretion (ADME) of whole *G. elegans* crude extract in female and male rats using high-performance liquid chromatography coupled with quadrupole time-of-flight mass spectrometry (HPLC/QqTOF-MS), which facilitates determining the reasons for the gender differences in toxicity. A total of 25 absorbed bioactive components and 3 related produced metabolites were tentatively identified in female rats, while only 17 absorbed bioactive components and 3 related produced metabolites were identified in male rats. By comparison of peak intensities, most compounds were found to be more active in absorption, distribution and excretion in female rats than in male rats, which showed that female rats were more sensitive to *G. elegans*. This study was the first to investigate the multicomponent in vivo process of *G. elegans* in rats and compare the differences between sexes. It was hypothesized that differences in the absorption of gelsedine-type alkaloids were one of the main reasons for the sex differences in *G. elegans* toxicity.

## 1. Introduction

Gelsemium is an evergreen woody vine of the genus Gelsemium of the family Loganiaceae. The genus is comprised of three species: the Asian *Gelsemium elegans* (*G. elegans*) and two North American species, *Gelsemium sempervirens* and *Gelsemium rankinii*. Phytochemical studies have shown that all species are rich in alkaloids and iridoterpenes. Indole alkaloids are the main active constituents and can be divided into six types according to their unique skeletons: gelsemine-type, gelsedine-type, sarpagine-type, humantenine-type, koumine-type and yohimbane-type [1,2,3]. As a traditional Chinese medicine (TCM), *G. elegans* is used to treat neuropathic pain, spasticity, rheumatoid arthritis, skin ulcers, and cancer [4,5]. North American Gelsemium is primarily used in homeopathic remedies [6]. Moreover, an increasing number of modern pharmacological studies have shown that *G. elegans* has affluent pharmacological activities, including antitumor, anti-inflammatory, analgesic, anxiolytic and immunomodulatory activities [7,8,9,10]. To date, some researchers have demonstrated the potential of Gelsemium as a treatment for COVID-19 [11,12,13]. All these results proved that Gelsemium has good potential for clinical development and application. Therefore, Gelsemium has attracted increasing attention worldwide.

However, Gelsemium is the world’s most famous poisonous plant. There are many cases of poisoning and death caused by *G. elegans* [14,15,16]. In fact, the development and application of *G. elegans* is limited by its toxicity. According to the reported the half lethal dose (LD_50_) of *G. elegans*, LD_50_ of different compounds varied greatly [1]. Gelsedine-type alkaloids could be determined to be the main toxic compounds. Gelsenicine [LD_50_ 0.128 mg/kg, mice (intraperitoneal injection (i.p.)); 0.26 mg/kg, rat (i.p.); 0.15 mg/kg, rat (intravenous injection (i.v.))] was the most toxic alkaloid in *G. elegans* and *Gelsemium sempervirens* [17]. In contrast, gelsemine was the most abundant compound [LD_50_ 56 mg/kg, mice, (i.p.)], and koumine was also a compound with high content and low toxicity [LD_50_ 99 mg/kg, mice, (i.p.)] [5]. According to toxicity reports, the LD_50_ of the crude alkaloid fraction lies between that of gelsenicine and gelsemine [LD_50_ 4 mg/kg, rat (i.p.); 15 mg/kg, rat (peros (p.o.))] [18]. This showed that the *G. elegans* extract contains these alkaloids, and the toxicity was mainly due to the presence of gelsedine-type alkaloids. Interestingly, related acute toxicity studies have shown that the toxicity of *G. elegans* differs between male and female rats [1]. Yang et al. found that 14-(R)-hydroxy-gelsenicine, a poisonous component of *G. elegans*, showed a significant gender difference with LD_50_ values of 0.125 mg/kg and 0.295 mg/kg for female and male rats, respectively [19].

The study of the absorption, distribution, metabolism, and excretion (ADME) of herbal medicines is very important for the elucidation of their physical basis of action. It had practical meaning to identify prototypes and metabolites in plasma and urine to reveal the functional material basis of TCMs. In addition, herbal medicine–mediated toxicity is often related to high exposure and accumulation of certain toxic components. Therefore, it is crucial to understand the distribution of each compound, especially toxic compounds, in different tissues. However, to date, a multicomponent study in rats and gender differences regarding the in vivo processes of the bioactive components of *G. elegans* have not been investigated, so this study aimed to elucidate the ADME in rats and the differences between female and male rats of the multiple components of *G. elegans*. Absorption and accumulation of bioactive ingredients in vivo played an important role in explaining and predicting efficacy and toxicity for *G. elegans*. In summary, this study improves the in vivo process study of *G. elegans* based on a previous study, and the improvement of the basic study of substance action will lay the foundation for its clinical development and application. In addition, the elucidation of sex differences in metabolites provides insights from new perspectives on the causes of toxicity differences.

## 2. Materials and Methods

### 2.1. Herb Material and Reagents

In total, 50 kg of the whole plant of *G. elegans* was collected from Fujian Province in China in June 2018. The samples were authenticated by Associate Professor Qi Tang at Hunan Agricultural University (National Center for Biotechnology Information Taxonomy ID: 427660). The dried *G. elegans* was milled into a powder, which was sealed and stored in a refrigerator at 4 °C. The powder was then passed through a 100-mesh sieve. The crude extract powder was then added to a saline solution to obtain a suspension, which was prepared promptly before oral administration to the rats.

Methanol, formic acid and acetonitrile were of chromatographic grade and obtained from Merck Chemicals Co. (Darmstadt, Germany). Water was prepared using a Milli-Q water purification system (Millipore, Bedford, MA, USA). All other reagents employed in the experiments were of analytical grade.

### 2.2. Animals and Administration

Three female and three male adult Sprague-Dawley rats (200 ± 20 g) were purchased from Hunan SJA Laboratory Animals Co., Ltd. (Changsha, China). The animal experiments were approved by the Ethics Committee of Hunan Agricultural University (Batch Number: 2020–43) and complied with the National Institute of Health (NIH) guidelines for the Care and Use of Laboratory Animals. Adult Sprague-Dawley rats were acclimatized to our laboratory conditions for 7 days with free access to a normal standard chow diet and tap water. The animals were kept under standard conditions of temperature (23 ± 2 °C) and relative humidity of 50%. After acclimatization for 7 days, the fine powder of *G. elegans* was added to a saline solution to obtain a suspension, given to rats through oral administration by gavage at a dose of 0.1 g/kg (*G. elegans* powder/body weight), which was set according to previously reported results [1]. The gavage volume of each rat was approximately 2 mL. Heparinized blood samples of rats were collected at 6.0 h following drug dosing. The blood was then centrifuged (3500× *g*, 15 min, 4 °C), and the supernatant was collected. Urine and fecal samples were collected 12–24 h post-dose. Intestinal tract samples, liver samples and kidney samples were collected when animals were killed by decapitation 24 h after dosing. Triplicate samples of each biological sample were prepared, and those samples were stored at −80 °C until analysis.

### 2.3. Sample Preparation

After frozen rat plasma/urine samples were naturally thawed to room temperature, 200.0 μL of plasma/urine was added to clean 2.0-mL centrifuge tubes. One milliliter of 1% formic acid-acetonitrile was used to precipitate proteins. The samples were vortexed fully and then centrifuged at 10,000 rpm for 10 min. The supernatant was filtered through a 0.22-μm microbore cellulose membrane and injected via an autosampler vial for HPLC/QqTOF-MS analysis.

Fecal and tissue samples were processed with reference to a previous study [20]. Briefly, feces and liver, kidney and intestinal tract samples (2 g) were accurately weighed and placed into clean 50.0-mL Eppendorf tubes. To better extract the drug components, the tissue could be homogenized before the extraction step. Four milliliters of 1% formic acid-acetonitrile were added to precipitate the proteins, and this step was repeated four times. The samples were vortexed fully and centrifuged at 10,000 rpm for 10 min. One milliliter of supernatant was filtered through a 0.22-μm microbore cellulose membrane and injected via an autosampler vial for HPLC/QqTOF-MS analysis.

### 2.4. Analysis Conditions for Metabolic Studies

An instrument coupled with an Agilent 6530 quadrupole time-of-flight mass spectrometer (Agilent Technologies, Santa Clara, CA, USA) was used for HPLC/QqTOF-MS analysis [21]. This system was equipped with an electrospray interface (ESI). The HPLC instrument consisted of a binary pump, an autosampler, an online degasser and a thermostatically controlled column compartment. The sample was separated by a Thermo-C18 column (2.1 mm × 150 mm I.D.; particle size 3.5 μm column). The mobile phase consisted of 0.1% formic acid (A) and acetonitrile (B). The gradient of the mobile phase was as follows: 0–2 min, 10% B; 2–7 min, 10–15% B; 7–25 min, 15–35% B; 25–32 min, 35–90% B; 32–35 min, 90% B; 35.01 min, 90–10% B; and 35.01–40 min, 10% B. Each sample was detected for 40 min. The flow rate was set to 0.3 mL/min. The column temperature was maintained at 30 °C.

The mass spectrometric detection conditions were set as follows: collection scope, 50–1000 *m*/*z*; capillary voltage, 4000 V; nebulizer pressure, 40 psi; drying gas flow rate, 9 L/min; gas temperature, 350 °C; octapole dc 1, 37.5 V; fragmentor voltage, 175 V; ctapolerf, 250 V; skimmer voltage, 60 V; and mode, positive ESI. The instrument performed the internal mass calibration automatically via an automated calibration delivery system. The calibrating solution displayed internal reference masses at *m*/*z* 121.0508 and 922.0098 in positive ion mode. Agilent Mass Hunter software (version B.01.03 Build 1.3.157.0 2) controlled all data acquisition. Additionally, peak intensities in this study were used to compare the relative concentrations of bioactive components.

## 3. Results

**Figure 1:**
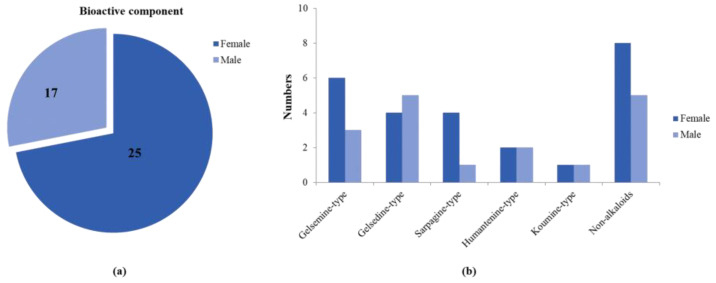
A comparative analysis of absorbed bioactive components in male and female rats. (**a**) The total amount of absorbed bioactive components in male and female rats. (**b**) The amount of absorbed bioactive components of different types in male and female rats.

In order to fully elucidate the material basis of the action of *G. elegans*, we comprehensively and systematically analyzed the exposure, metabolism and disposal of multiple components of *G. elegans* in urine, plasma, feces and tissue samples. Profiling and characterization of *G. elegans* bioactive components and metabolite components in rat samples mainly referred to previous strategies and results [20]. The extract ion chromatogram (EIC) was extracted to observe precise molecular masses, and retention time was compared with previous experiments [20]. In addition, the verification of MS/MS spectra greatly increased the confidence of the identification results. All of these compounds’ information has been summarized in Table 1, including structure, molecular formula, retention time, observed mass and other relevant information. The number of identified bioactive components is shown in Figure 1. We attempted to identify compounds other than the bioactive components detected in goats by using an established *G. elegans* database or summarized the cleavage pattern of different types of *G. elegans* alkaloids [3,21]. Unfortunately, we were unable to identify more new bioactive components because the concentrations were so low that no MS/MS data were available.

### 3.1. Comparative Analysis of Urine Samples from Male and Female Rats

In female rat urine samples, through the analysis of the original MS data, a total of 15 natural product components were identified, including five gelsemine-type alkaloids (compounds H1~H5), three gelsedine-type alkaloids (compounds H7, H8, and H11), two sarpagine-type alkaloids (compounds H12, H13), one humantanine-type alkaloid (compound H16), one koumine-type alkaloid (compound H18), two iridoids (compounds H21 and H23) and one triterpene (compound H27). In addition, two metabolites, H6-M1 and H6-M2, were detected in the urine samples, which were tentatively identified as dehydrogenation and oxidation metabolites, respectively.

In male rat urine samples, a total of ten natural product compositions were identified, including three gelsemine-type alkaloids (compounds H1, H3, H5), two gelsedine-type alkaloids (compounds H7 and H11), one humantanine-type alkaloid (compound H16), one koumine-type alkaloid (compound H18), two iridoids (compounds H21 and H23) and one triterpene (compound H27). In addition, two compound-related oxidation metabolites, H2-M2 and H3-M1, were identified in male rat urine samples.

By comparing peak intensities of all jointly characterized bioactive components, we found higher concentrations in all female rat urine samples than in male rats. This result indicated that the excretion of the bioactive components of *G. elegans* was faster and more abundant in female rats than in male rats.

### 3.2. Comparative Analysis of Plasma Samples from Male and Female Rats

As shown in Table 1, nine natural products were identified by the analysis of female rat plasma samples, including three gelsemine-type alkaloids (compounds H1, H2 and H6), one gelsedine-type alkaloid (compound H8), one koumine-type alkaloid (compound H18), and four iridoids (compounds H19~H22). In addition, the dehydrogenation metabolite H6-M1 of compound H6 was detected.

In male rat plasma samples, a total of five natural product compositions were identified, including one gelsemine-type alkaloid (compound H2), one gelsedine-type alkaloid (compound H8), one koumine-type alkaloid (compound H18) and two iridoids (compounds H21 and H22). The compounds identified in the plasma samples were much less abundant than those in the urine, indicating that compounds of *G. elegans* were easily and rapidly eliminated and excreted in rats.

Only the absorbed chemical components or related metabolites in blood, which maintained a certain concentration in target organs for a finite period of time, were responsible for the therapeutic effects. The comparison of peak intensities of *G. elegans*-related bioactive components revealed that the levels of the same component in the plasma of male and female rats did not differ significantly. Additionally, combined with the results of the above urine samples, it was assumed that the bioactive components absorbed by the female rats were distributed to the tissues or excreted into the urine at a faster rate.

### 3.3. Comparative Analysis of Fecal Samples from Male and Female Rats

Through the analysis of female feces samples, a total of ten natural product components were identified, including four gelsemine-type alkaloids (compounds H1~H3 and H5), one gelsedine-type alkaloid (compound H9), one sarpagine-type alkaloid (compound H14), two humantanine-type alkaloids (compounds H16 and H17), one iridoid (compound H22) and one triterpene (compound H27). In addition, the oxidation metabolite H2-M2 was detected in fecal samples from female rats.

In male rat feces samples, a total of six natural product compositions were identified, including two gelsemine-type alkaloids (compounds H3 and H5), one gelsedine-type alkaloid (compound H9), one humantanine-type alkaloid (compound H17) and two iridoids (compounds H21 and H22). No related metabolites were detected in male fecal samples.

By comparing the relative peak intensities of the prototype components in the feces samples of female and male rats, it was found that they were slightly higher in females than in males. We know that most of the chemical components in feces are unabsorbed by the animal, and some might be absorbed and metabolized compounds excreted through the hepatic and intestinal circulation. The slightly higher content of bioactive components in fecal samples in female rats might be explained by the hepatic-intestinal circulation of the organism.

### 3.4. Comparative Analysis of Tissue Samples from Male and Female Rats

Through the analysis of female intestinal tract samples, a total of four natural product components were identified, including one sarpagine-type alkaloid (compound H15), one koumine-type alkaloid (compound H18) and two iridoids (compounds H25 and H26). Meanwhile, a total of two natural product components were identified in male intestinal tract samples, including one gelsedine-type alkaloid (compound H10) and one koumine-type alkaloid (compound H18). In addition, the metabolite H2-M1 was detected as a glucuronidation metabolite in male intestinal tract samples. However, no metabolites were detected in female intestinal tract samples.

In female liver samples, a total of nine natural product components were identified, including one gelsemine-type alkaloid (compound H2), two gelsedine-type alkaloids (compounds H8 and H9), one sarpagine-type alkaloid (compound H13), two humantanine-type alkaloids (compounds H16 and H17) and three iridoids (compounds H21, H22 and H26). Meanwhile, a total of six natural product components were identified in male liver samples, including two gelsedine-type alkaloids (compounds H8 and H9), one humantanine-type alkaloid (compound H17), one koumine-type alkaloid (compound H18) and two iridoids (compounds H21 and H26). No related metabolite was detected in either female or male liver samples.

Through the analysis of female kidney samples, a total of five natural product components were identified, including one gelsemine-type alkaloid (compound H2), one sarpagine-type alkaloid (compound H13), one humantanine-type alkaloid (compound H17), one koumine-type alkaloid (compound H18) and one iridoid (compound H21). Meanwhile, a total of seven natural product components were identified in male kidney samples, including one gelsemine-type alkaloid (compound H2), one gelsedine-type alkaloid (compound H9), one humantanine-type alkaloid (compound H17), one koumine-type alkaloid (compound H18), two iridoids (compounds H21 and H26) and one triterpene (compound H27). No related metabolite was detected in either female or male kidney samples.

Based on the peak intensities of the prototype compounds in different tissues, we found that the content of bioactive components of *G. elegans* was higher in both liver and kidney tissues of female rats than in males. However, fewer prototype compounds were characterized in the intestinal tract samples, and there was no clear pattern from their peak intensities. Through the analysis of the tissue samples, first, the components of *G. elegans* were widely distributed. In addition, the fewer kinds of compounds detected in the tissue samples indicated that many *G. elegans* compounds were eliminated in a short time.

## 4. Discussion

Previously, the authors studied the absorption and metabolism of *G. elegans* multicomponents in goat plasma, urine and feces samples. The present study used the same sample processing methods and mass spectrometry detection conditions to investigate the metabolism of multiple components of *G. elegans* in female and male rats. Therefore, based on a previous study, we did not carry out an analysis of the cleavage law for the compounds already identified. All of these compounds’ information has been summarized in Table 1. We attempted to identify compounds other than the natural product detected in goats by using an established *G. elegans* database or summarized the cleavage pattern of different types of *G. elegans* alkaloids [3,21]. Unfortunately, however, we were unable to identify more new natural products because the concentrations were so low that no MS/MS data were available.

Although ADME research on several standards of *G. elegans* has been carried out in rats [22,23,24,25], these studies have greatly facilitated the screening and identification of bioactive ingredients of *G. elegans*. However, the overall administration of *G. elegans* was a synergistic result of multiple components, and there were many interactions. Therefore, a holistic and sexed study of the in vivo processes of *G. elegans* is essential. This study was the first to investigate gender differences in the multicomponent in vivo processes of *G. elegans*. A total of 27 bioactive components and 3 related metabolites were tentatively identified in male and female rats, and the amounts of absorbed bioactive components in male and female rats are shown in Figure 1a,b. At the same time, we took the peak intensity of the compound as a reference for the concentration of the compound. Overall, female rats that absorbed compounds of *G. elegans* were more active and had a wider distribution than male rats, and it was also faster to eliminate.

The composition of *G. elegans* is rich and complex, including indole alkaloids, iridoids, and steroids [3,21]. Indole alkaloids are the major active components in *G. elegans* and are grouped into six types according to their unique skeletons. In this study, all classes of alkaloids except yohimbane-type were detected and identified. In addition, the prototypes and metabolites detected in this study were less than those of goats and pigs that had been reported [20,26]. It was speculated that on the one hand, the dosage was small, and on the other hand, the sampling time was relatively long from the administration time. Both of these lead to low concentrations of the compound in the body. Very few metabolites were detected in this study, which may be because even if the relevant metabolites were produced, the concentration was too low to reach the detection threshold of the instrument.

**Figure 2:**
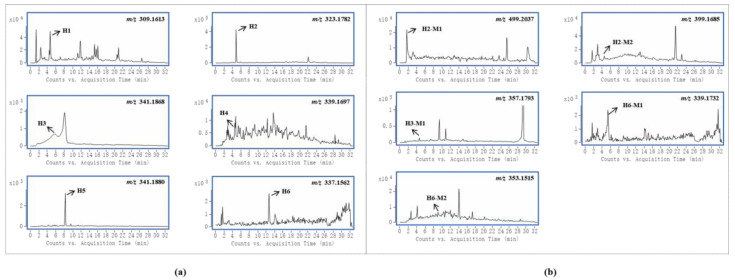
Accurate extracted ion chromatograms (EICs) of natural products of gelsemine alkaloids (**a**) and their metabolites (**b**) obtained from samples.

Gelsemine-type alkaloids are the most abundant and least toxic in *G. elegans* [1]. In this study, six and four gelsemine-type bioactive components were absorbed by female and male rats, respectively. The EICs of these alkaloids and their metabolites obtained from samples are shown in Figure 2a,b, respectively. By comparing the peak intensities, it was found that the prototypes showed higher intensities in the samples from female rats. This suggests that female rats were more active in the absorption of these alkaloids, both in terms of the amount and concentration of compounds. In addition, it was evident that gelsemine-type bioactive components were more widely distributed in female rats. Due to the abundance of this class of alkaloids and the high level of exposure, the five metabolites identified in the study were all from the biotransformation of these alkaloids, including three oxidized metabolites, one hydrogenated metabolite and one glucuronidated metabolite. Certainly, in goats, more gelsemine-type bioactive component-relevant metabolites were detected and identified, as evidenced by the presence of more glucuronidated metabolites and dehydrogenated metabolites [20]. Moreover, the hypothesis that active phase II metabolism could reduce toxicity was proposed in our group’s previous study about *G. elegans* multicomponent metabolism in goats [20]. This corroborated the results of the current study. As seen in this study, the only phase II metabolite was detected in intestinal tract samples of male rats, but no metabolites were detected in female rats.

**Figure 3:**
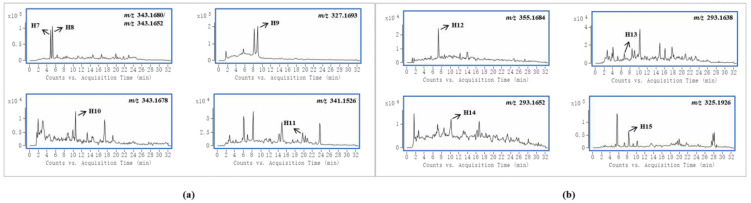
An accurate extracted ion chromatograms (EICs) of gelsedine-type alkaloids (**a**) and sarpagine-type alkaloids (**b**).

Gelsedine-type alkaloids are the most toxic alkaloids in *G. elegans,* and their presence is a major factor in the toxicity of *G. elegans* [1]. In this study, four and five prototypes were identified in female and male rats, respectively, and the EICs of these alkaloids are shown in Figure 3a. By analyzing the peak intensities of these prototypes in different samples, we found that the peak intensities of most compounds were higher in female samples. This suggested that these toxic components were more highly exposed in female rats. In particular, compound H9 was the most toxic alkaloid in *G. elegans* [27], and it existed in higher concentrations in female rat plasma samples than in male rats, which was consistent with the published pharmacokinetic results of gelsenicine in male and female rats [28]. In previous studies on the toxicity of *G. elegans* in rats, a parenteral solution of crude alkaloidal extract was given to rats via intraperitoneal injection, and the LD_50_ of female and male rats was 1.2 mg/kg and 1.5 mg/kg, respectively [1]. 14-(R)-hydroxy-gelsenicine was proven to have a significant gender difference with LD_50_ values of 0.125 mg/kg and 0.295 mg/kg for female and male mice, respectively [28]. In Li et al.’s acute toxicity study, gelsenicine was highly toxic, and female rats exhibited greater sensitivity to gelsenicine than male rats (LD_50_ 0.520 mg/kg vs. 0.996 mg/kg, respectively) [28]. These results indicated that female rats were more sensitive to the toxic components of *G. elegans* and there was significantly more toxic to female rats than male rats. Through the analysis of gender differences in the in vivo processes of the multiple components of *G. elegans*, it was found that more alkaloid components were identified in female rats than in males, and the peak concentrations were mostly higher. We speculated that one of the reasons for the above gender differences in toxicity was that female rats had a stronger and faster absorption of *G. elegans* bioactive compounds, and gelsedine-type alkaloids played a major role in this process. It is also worth mentioning that these bioactive components were present in high levels in urine and relatively little in plasma and tissues, indicating their rapid excretion. Previous subchronic toxicity studies have shown that *G. elegans* does not cause accumulation damage in the organism, which is inseparable from the rapid excretion of the main toxic components [29]. Cytochrome P450 enzymes (CYP450) are widely distributed in the body and play pivotal roles in drug metabolism [30]. Some studies have shown that CYP3A4 is the main metabolic enzyme of gelsedine-type alkaloids [31,32]. It is worth noting that the types and activities of these enzymes differ among sex, which results in differences in drug metabolism [33]. These differences may affect their in vivo bioactivities and toxicities [34]. Therefore, the sex differences of metabolic enzyme would be an important reason to explain sex differences in the toxicity of *G. elegans*.

Sarpagine-type alkaloids had the most significant gender differences in this study, and four and one alkaloid were detected in female and male rats, respectively, whose EICs are shown in Figure 3b. The peak intensity of the co-detected compound H12 was higher in female rats. Two kinds of humantenine-type alkaloids were detected in both female and male rats, and the differences were that the distribution in female rats was more extensive and the content was higher. Their EICs are shown in Figure S1A. Koumine (H18) was widely distributed in each sample of female and male rats, and its EIC is shown in Figure S1B. Notably, the peak intensity of koumine in female rat urine samples was nearly three times that of male rats, but it was present at lower levels in female rat tissue samples. This indicated that koumine was eliminated faster in female rats than in male rats.

In addition to alkaloids, there are many nonalkaloid components in *G. elegans*, such as iridoids and triterpenes [35]. Eight and five nonalkaloid compounds were detected in female and male rats, respectively, which suggested that female rats absorbed these compounds more easily. Their EICs are shown in Figure S2. In addition, we also found that most of the compounds were more abundant in female rat urine and feces samples and lower in plasma and tissue samples than in male rats. It could be speculated that the nonalkaloid components will be excreted faster in female rats. It has been reported that nonalkaloid compounds have antitumor pharmacological activities [36]. These highly absorbable bioactive nonalkaloids contribute to the pharmacological activity of *G. elegans*, such as anti-cancer [37].

In addition to sex differences, there are significant species differences in *G. elegans* toxicity. The active ingredients of *G. elegans* mainly show pharmacological activity with less toxicity in pigs and goats, while it is highly toxic to rats and mice [20,28]. Multi-component metabolic studies of *G. elegans* in both goats and pigs indicated that the phase II metabolic reaction of glucuronidation was its most active metabolic way [20,28], while only one glucuronidated metabolite in rats. It was speculated that the difference in compound metabolism was one of the reasons leading to the difference in toxicity. In addition, active phase II metabolism might be beneficial to the reduction in toxicity in animals.

## 5. Conclusions

A multicomponent in vivo process study of *G. elegans* in rats was studied for the first time. In this study, HPLC/QqTOF-MS was used to detect drugs as a sensitive and reliable method, and we identified or tentatively characterized a total of 25 absorbed bioactive components and 3 related produced metabolites in female rats, while only 17 absorbed bioactive components and 3 related produced metabolites in male rats. Combining the peak intensity data of the compounds, we found that the absorption, distribution and excretion of *G. elegans* alkaloids in female rats were more active than those in male rats, specifically the gelsedine-type alkaloids. In brief, there were significant gender differences in the in vivo process of *G. elegans* in rats, which provided strong evidence for gender differences in *G. elegans* toxicity. It was hypothesized that differences in the absorption of gelsedine-type alkaloids were one of the main reasons for the sex differences in *G. elegans* toxicity. In summary, on the basis of the previous study, this study further improved the in vivo exposure process of *G. elegans*, which laid an experimental foundation for the development and application of *G. elegans*. At the same time, it is of great significance to clarify the mechanism of toxicity and the reasons for the difference in toxicity.

## Figures and Tables

**Table 1:** The compound class, skeletons, name, structure, retention time (RT), formula, observed mass, mass error, and biotransformation of natural products and their metabolites in samples as detected by HPLC/QqTOF-MS.

No.	Compound Class	Structures	Name	Groups/Proposed Metabolism	RT	Molecular Formula	[M + H]^+^ (*m*/*z*)	Error (ppm)	Rats (Female)	Rats (Male)
1	Alkaloid, Gelsemine-type	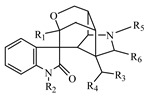	Unknown(H1)	R_1_ = R_2_ = R_4_ = R_5_ = R_6_ = H, R_3_ = CH_2_	4.668	C_19_H_20_N_2_O_2_	309.1613	−5.02	U (5.0 × 10^4^) P (1.5 × 10^3^) F (1.2 × 10^4^)	
2			Gelsemine(H2)	R_1_ = R_2_ = R_4_ = R_6_ = H, R_3_ = R_5_ = CH_2_	4.867	C_20_H_22_N_2_O_2_	323.1782	−8.68	U (5.2 × 10^5^) P (4.0 × 10^3^) F (3.2 × 10^4^) L (4.0 × 10^4^) K (2.0 × 10^4^)	U (1.0 × 10^4^) P (8.0 × 10^2^) K (1.5 × 10^4^)
3			H2-M1	+GlcA	1.625	C_26_H_30_N_2_O_8_	499.2037	7.61		I (1.3 × 10^4^)
5			H2-M2	+O	4.570	C_20_H_22_N_2_O_3_	339.1685	5.38	F (9.0 × 10^3^)	U (1.2 × 10^4^)
6			19R-Hydroxydihydrogelsemine(H3)	R_1_ = R_2_ = R_6_ = H, R_3_ = OH, R_4_ = R_5_ = CH_3_	5.269	C_20_H_24_N_2_O_3_	341.1868	−2.44	U (2.0 × 10^4^) F (8.0 × 10^4^)	U (1.0 × 10^4^) F (3.5 × 10^4^)
8			H3-M1	+O	4.668	C_20_H_24_N_2_O_4_	357.1793	4.45		U (8.0 × 10^3^)
9			Gelsemine Oxide(H4)	R_1_ = R_2_ = R_4_ = R_6_ = H, R_3_ = CH_3_ oxide, R_5_ = CH_3_	5.178	C_20_H_22_N_2_O_3_	339.1697	1.83	U (1.2 × 10^4^)	
10			19S-Hydroxydihydrogelsemine(H5)	R1 = R2 = R6 = H, R_3_ = OH, R_4_ = R_5_ = CH_3_	8.184	C_20_H_24_N_2_O_3_	341.1880	−5.97	U (3.0 × 10^5^) F (4.0 × 10^5^)	U (1.4 × 10^5^) F (1.6 × 10^5^)
11			21-Oxogelsemine(H6)	R_1_ = R_2_ = R_4_ = H, R_4_ = CH_2_, R_5_ = CH_3_, R_6_ = O	12.707	C_20_H_20_N_2_O_3_	337.1562	−4.55	P (3.0 × 10^3^)	
12			H6-M1	+2H	5.472	C_20_H_22_N_2_O_3_	339.1732	−8.52	U (7.5 × 10^3^) P (1.5 × 10^3^)	
13			H6-M2	+O	9.088	C_20_H_20_N_2_O_4_	353.1515	−5.44	U (7.0 × 10^3^)	
14	Alkaloid, Gelsedine-type	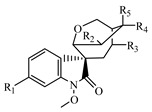	11-Hydroxygelsenicine(H7)	R_1_ = OH, R_2_ = R_5_ = H, R_3_ = N, R_4_ = C(CH_2_CH_3_)	4.869	C_19_H_22_N_2_O_4_	343.1680	−8.08	U (1.0 × 10^5^)	U (1.0 × 10^4^)
15			14-Hydroxygelsenicine(H8)	R_1_ = R_5_ = H, R_2_ = OH, R_3_ = N, R_4_ = C(CH_2_CH_3_)	5.269	C_19_H_22_N_2_O_4_	343.1652	0.10	U (1.2 × 10^5^) P (1.0 × 10^3^) L (1.3 × 10^4^)	P (3.0 × 10^3^) L (3.8 × 10^4^)
16			Gelsenicine(H9)	R_1_ = R_2_ = R_4_ = H, R_3_ = N, R_5_ = CH_3_	8.990	C_19_H_22_N_2_O_3_	327.1693	2.82	F (2.1 × 10^5^) L (4.0 × 10^4^)	F (6.0 × 10^4^) L (8.0 × 10^3^) K (1.2 × 10^4^)
17			Hydroxyl of gelsenicine(H10)	unknown	10.598	C_19_H_22_N_2_O_4_	343.1678	−7.10		I (1.3 × 10^4^)
18			19-Oxogelsencine(H11)	R_1_ = R_2_ = R_5_ = H, R_3_ = N, R_4_ = C(COCH_3_)	19.743	C_19_H_20_N_2_O_4_	341.1526	−8.87	U (2.5 × 10^4^)	U (1.1 × 10^4^)
19	Alkaloid, Sarpagine-type	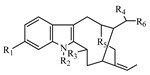	Unkonw(H12)	unknown	7.279	C_20_H_22_N_2_O_4_	355.1684	−8.94	U (3.0 × 10^4^)	U (1.3 × 10^4^)
20			Dehydrokoumidine(H13)	R_1_ = R_2_ = R_3_ = R_6_ = H, R_4_ = O, R_5_ = N	6.676	C_19_H_20_N_2_O	293.1638	3.56	U (1.4 × 10^4^) L (2.8 × 10^4^) K (8.0 × 10^3^)	
21			Unknown(H14)	Unknown	10.196	C_19_H_20_N_2_O	293.1652	−1.23	F (1.3 × 10^4^)	
22			Gardnerine(H15)	R_1_ = OCH_3_, R_2_ = R_3_ = R_6_ = H, R_4_ = CH_2_OH, R_5_ = N	8.286	C_20_H_24_N_2_O_2_	325.1926	−4.77	I (7.0 × 10^4^)	
23	Alkaloid, Humantenine-type	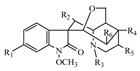	14-Hydroxyrankinidine(H16)	R_1_ = R_2_ = R_3_ = R_4_ = H, R_5_ = CHCH_3_, R_6_ = OH	10.898	C_20_H_24_N_2_O_4_	357.1813	−1.17	U (4.0 × 10^4^) F (4.0 × 10^4^) L (7.0 × 10^3^)	U (2.5 × 10^4^)
24			Humantenine(H17)	R_1_ = R_2_ = R_4_ = R_6_ = H, R_3_ = CH_3_, R_5_ = CHCH_3_	13.613	C_21_H_26_N_2_O_3_	355.2034	−5.03	F (1.5 × 10^4^) L (2.5 × 10^4^) K (1.0 × 10^4^)	F (1.0 × 10^4^) L (3.0 × 10^3^) K (7.0 × 10^3^)
25	Alkaloid, Koumine-type	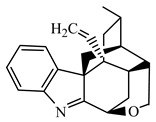	Koumine(H18)	\	6.475	C_20_H_22_N_2_O	307.1817	−3.95	U (2.8 × 10^4^) P (8.0 × 10^2^) I (3.5 × 10^4^)K (1.0 × 10^4^)	U (1.0 × 10^4^) P (7.0 × 10^2^) I (6.0 × 10^4^) L (6.0 × 10^3^) K (1.3 × 10^4^)
26	Iridoids	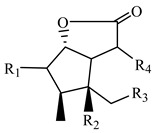	Geleganoid A/GRIR-1(H19)	R_1_ = R_2_ = OH, R3, R_4_ = OCH(OH)	1.657	C_10_H_14_O_6_	231.0878	−6.46	P (1.5 × 10^4^)	
27			7-Hydroxygelsemiol/9- Hydroxygelsemiol(H_2_O)	R_1_ = H, R_2_ = R_3_ = OH, R_4_ = CH_2_OH	2.159	C_10_H_16_O_5_	217.1050	9.49	P (2.6 × 10^4^)	
28			9-Hydroxysemperoside(H21)	R_1_ = H, R_2_ = OH, R_3_, R_4_ = OCH(OCH_2_CHOHC_4_H_7_O_4_)	5.872	C_16_H_24_O_10_	377.1468	−6.85	U (7.2 × 105) P (7.0 × 102) L (1.5 × 106) K (1.1 × 106)	U (5.0 × 105) P (7.0 × 102) F (3.0 × 104) L (1.3 × 106) K (1.5 × 106)
29			GSIR-1(H22)	R_1_ = R_2_ = H, R_3_ = OH, R_4_ = CH_2_	3.862	C_10_H_14_O_3_	183.1027	−6.20	P (1.2 × 103) F (7.0 × 103) L (7.0 × 103)	P (1.5 × 103) F (6.0 × 103)
30			Unknow(H23)	Unknown	3.058	C_10_H_12_O_3_	171.1011	2.77	U (1.2 × 104)	
31			7-Deoxygelsemide/9-Deoxygelsemide(H24)	R_1_/R_2_ = OH, R2_/_R_1_ = H, R_3_, R_4_ = OCH	4.969	C_10_H_12_O_4_	197.0825	−8.49		U (1.0 × 104)
32			9-DeoxyGRIR-2(H25)	R_1_ = R_2_ = H, R_3_, R_4_ = OCH(OH)	6.276	C_10_H_14_O_4_	199.0947	9.01	I (2.5 × 104)	
33			Isomer of 7-Deoxygelsemide/9-Deoxygelsemide(H26)	R_1_/R_2_ = OH, R_2_/R_1_ = H, R_3_ = OH, R_4_ = CH_2_OH	6.678	C_10_H_16_O_5_	217.1078	−3.47	I (4.0 × 104) L (6.0 × 103)	L (5.0 × 103) K (5.0 × 104)
34	Triterpene	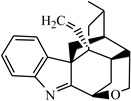	3-keto-urs-11-en-13β(28)-olide(H27)	Unknown	16.526	C_30_H_44_O_3_	453.3396	−7.25	U (3.0 × 10^4^) F (6.0 × 105)	U (2.0 × 104) K (1.4 × 105)

“H” means Bioactive components; “M” means metabolites (M1 to M3 means the natural product had three metabolites identified); “U” means urine sample; “P” means plasma sample; “F” means feces sample; “I” means intestinal tract sample; “L” means liver sample; “K” means kidney sample; “()” means peak intensity of EIC.

## Data Availability

Not applicable.

## Supplementary Materials

The following supporting information can be downloaded at: https://www.mdpi.com/article/10.3390/metabo13010033/s1, Figure S1: Accurate extracted ion chromatograms (EICs) of humantenine-type alkaloids (a) and koumine (b). Figure S2: Accurate extracted ion chromatograms (EICs) of nonalkaloids.
