# Supplementary material for: Sex Differences in the In Vivo Exposure Process of Multiple Components of Gelsemium elegans in Rats
Source: Metabolites. 2022 Dec 24;13(1):33. doi: 10.3390/metabo13010033 (PMC9865510; doi:10.3390/metabo13010033)
Supplement: Supplementary file 1 [file metabolites-13-00033-s001.zip › metabolites-2084137-supplementary.pdf]

Figure S1. Accurate extracted ion chromatograms (EICs) of humantenine-type alkaloids (a) and koumine (b).

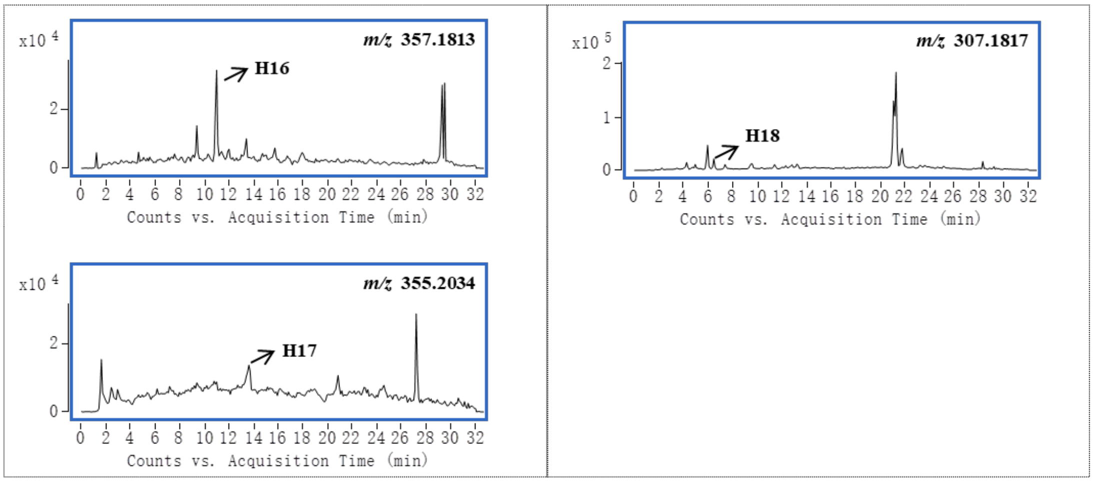

(a) **(b)**

Figure S2. : Accurate extracted ion chromatograms (EICs) of nonalkaloids.

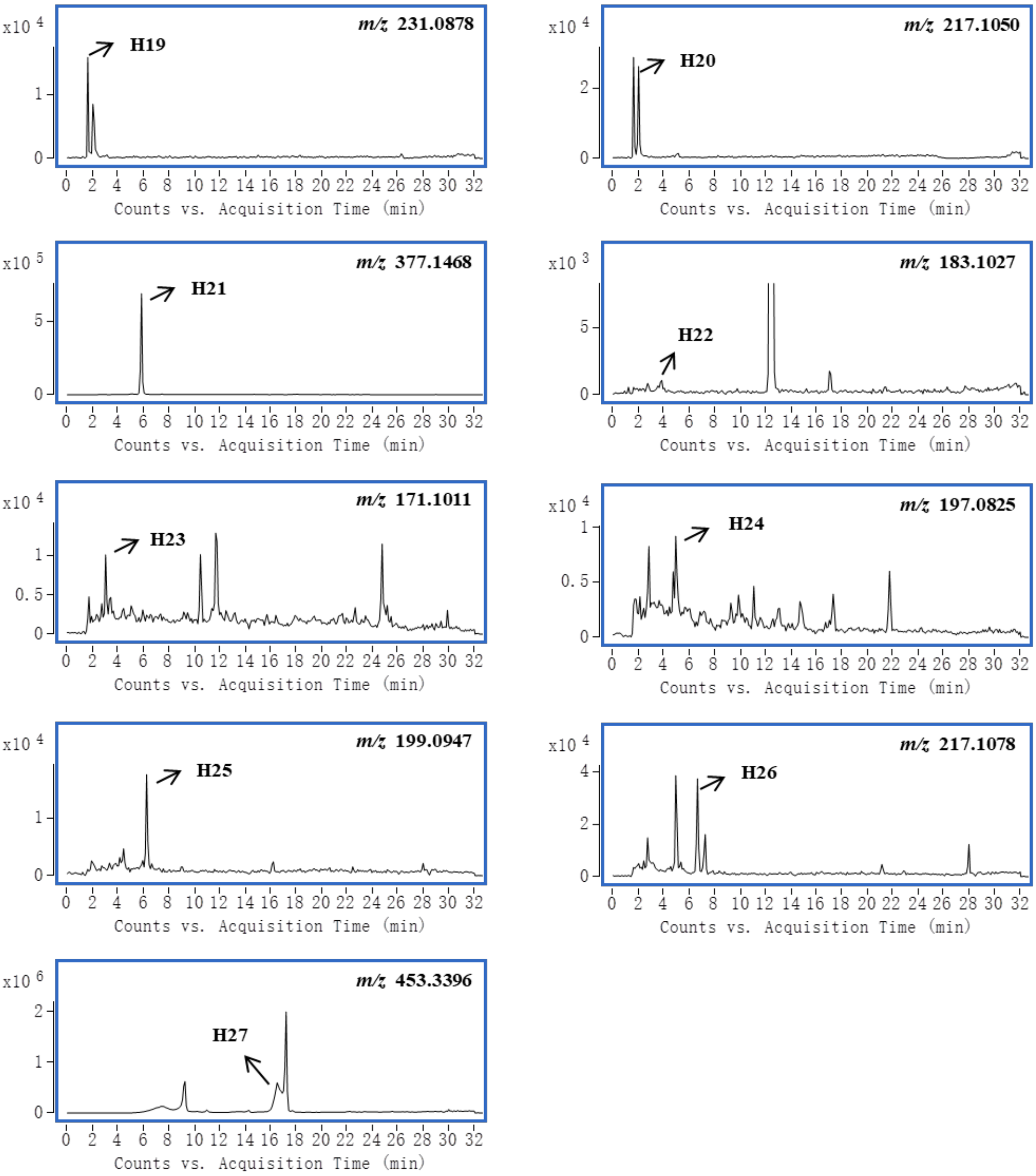

x10 4
m/z 231.0878
H19
1
0
0 2 4 6 8 10 12 14 16 18 20 22 24 26 28 30 32
Counts vs. Acquisition Time (min)
x10 4
m/z 217.1050
H20
2
0
0 2 4 6 8 10 12 14 16 18 20 22 24 26 28 30 32
Counts vs. Acquisition Time (min)
x10 5
m/z 377.1468
H21
5
0
0 2 4 6 8 10 12 14 16 18 20 22 24 26 28 30 32
Counts vs. Acquisition Time (min)
x10 3
m/z 183.1027
H22
5
0
0 2 4 6 8 10 12 14 16 18 20 22 24 26 28 30 32
Counts vs. Acquisition Time (min)
x10 4
m/z 171.1011
H23
1
0.5
0
0 2 4 6 8 10 12 14 16 18 20 22 24 26 28 30 32
Counts vs. Acquisition Time (min)
x10 4
m/z 197.0825
H24
1
0.5
0
0 2 4 6 8 10 12 14 16 18 20 22 24 26 28 30 32
Counts vs. Acquisition Time (min)
x10 4
m/z 199.0947
H25
1
0
0 2 4 6 8 10 12 14 16 18 20 22 24 26 28 30 32
Counts vs. Acquisition Time (min)
x10 4
m/z 217.1078
H26
4
2
0
0 2 4 6 8 10 12 14 16 18 20 22 24 26 28 30 32
Counts vs. Acquisition Time (min)
x10 6
m/z 453.3396
H27
2
1
0
0 2 4 6 8 10 12 14 16 18 20 22 24 26 28 30 32
Counts vs. Acquisition Time (min)
